# Prader-Willi Critical Region, a Non-Translated, Imprinted Central Regulator of Bone Mass: Possible Role in Skeletal Abnormalities in Prader-Willi Syndrome

**DOI:** 10.1371/journal.pone.0148155

**Published:** 2016-01-29

**Authors:** Ee-Cheng Khor, Bruce Fanshawe, Yue Qi, Sergei Zolotukhin, Rishikesh N. Kulkarni, Ronaldo F. Enriquez, Louise Purtell, Nicola J. Lee, Natalie K. Wee, Peter I. Croucher, Lesley Campbell, Herbert Herzog, Paul A. Baldock

**Affiliations:** 1 Bone and Mineral Research Division, Garvan Institute of Medical Research, Darlinghurst, Sydney, NSW, Australia; 2 Neuroscience Division, Garvan Institute of Medical Research, Darlinghurst, Sydney, NSW, Australia; 3 Diabetes and Obesity Research Division, Garvan Institute of Medical Research, Darlinghurst, Sydney, NSW, Australia; 4 School of Medical Sciences, University of NSW, Kensington, Sydney, NSW, Australia; 5 Department of Pediatrics, College of Medicine, Center for Smell and Taste, University of Florida, Gainesville, Florida, United States of America; University of Münster, GERMANY

## Abstract

Prader-Willi Syndrome (PWS), a maternally imprinted disorder and leading cause of obesity, is characterised by insatiable appetite, poor muscle development, cognitive impairment, endocrine disturbance, short stature and osteoporosis. A number of causative loci have been located within the imprinted Prader-Willi Critical Region (PWCR), including a set of small non-translated nucleolar RNA’s (snoRNA). Recently, micro-deletions in humans identified the snoRNA Snord116 as a critical contributor to the development of PWS exhibiting many of the classical symptoms of PWS. Here we show that loss of the PWCR which includes Snord116 in mice leads to a reduced bone mass phenotype, similar to that observed in humans. Consistent with reduced stature in PWS, PWCR KO mice showed delayed skeletal development, with shorter femurs and vertebrae, reduced bone size and mass in both sexes. The reduction in bone mass in PWCR KO mice was associated with deficiencies in cortical bone volume and cortical mineral apposition rate, with no change in cancellous bone. Importantly, while the length difference was corrected in aged mice, consistent with continued growth in rodents, reduced cortical bone formation was still evident, indicating continued osteoblastic suppression by loss of PWCR expression in skeletally mature mice. Interestingly, deletion of this region included deletion of the exclusively brain expressed Snord116 cluster and resulted in an upregulation in expression of both NPY and POMC mRNA in the arcuate nucleus. Importantly, the selective deletion of the PWCR only in NPY expressing neurons replicated the bone phenotype of PWCR KO mice. Taken together, PWCR deletion in mice, and specifically in NPY neurons, recapitulates the short stature and low BMD and aspects of the hormonal imbalance of PWS individuals. Moreover, it demonstrates for the first time, that a region encoding non-translated RNAs, expressed solely within the brain, can regulate bone mass in health and disease.

## Introduction

Prader-Willi syndrome (PWS), initially described in 1956 [1], is a neurological disorder with a number of clinical symptoms that greatly impair an individual’s standard of living, most characteristically excessive hunger and marked obesity. The main features of the PWS phenotype are more broad, and include: hypotonia, hyperphagia, hypogonadism, motor development issues, cognitive impairment, growth hormone (GH) deficiency, dysmorphic features and behavioural issues [2]. Importantly, PWS patients also exhibit a marked bone phenotype, with reduced stature, low bone mineral density (BMD) and scoliosis [3–5].

The genetic basis for PWS results from the inactivation of the Prader-Willi Critical Region (PWCR), q11-q13 region on paternal chromosome 15 [6], which contains a cluster of imprinted genes. The PWCR region on the maternal chromosome is imprinted and therefore epigenetically silenced through methylation, leading to mono-allelic expression of the paternal genes [6]. Interstitial deletions (65–70%), maternal uniparental disomy (20–30%) and mutations within the imprinting centre (2–5%) account for most PWS cases [2, 6]. Adding to the complexity, the PWCR contains several clusters of C/D box snoRNAs (small nucleolar RNAs) [7–10], which are located within the introns of the *SNURF-SNRPN* gene, which resides in this region. These snoRNA transcripts get processed and released during the maturation of the SNURF-SNRPN transcript. One of these snoRNA clusters in humans is the *HBII-85* (*SNORD116*) region containing 29 tandem repeats of *SNORD116* that are widely expressed in the brain [11]. SnoRNAs gain functionality as a result of protein association to give rise to small nuclear ribonucleoproteins (snRNPs), which interact with target RNAs through base pair interactions.

The targets of the snoRNA, *SNORD116*, remain elusive; however it has been implicated in the aetiology of PWS. Several cases of microdeletion only affecting the *SNORD116* cluster have been identified in humans, all of which gave rise to aspects of the classical clinical manifestations of PWS [6]. Importantly, PWCR knockout mice bear many of the phenotypic symptoms of PWS individuals like the significant increase in food intake so typical for PWS [12]. However, to date the effect of loss of the PWCR on skeletal tissue has not been determined.

Prepubertal PWS children have normal BMD (when adjusted for their short stature) [13–15], but during adolescence and adulthood, they have reduced total BMD and bone mineral content (BMC) likely due to lack of bone mineral accrual [3–5]. As a result, osteoporosis is prevalent in PWS individuals, who also face other orthopaedic complications associated with their weight gain, including scoliosis, kyphosis, hip dysplasia and fractures [4, 16]. The causes for this reduced BMD have been linked to reduced levels of growth hormone (GH) and hypogonadism [4]. However, GH treatment on PWS children did not increase BMD despite increases in height SD-scores, indicating that other factors are involved [13]. Further studies are required to investigate the cause of the PWS skeletal phenotype including the genes known to be involved in PWS such as *Snord116*. The aim of this study was therefore to examine the effects of PWCR deficiencies on bone homeostasis in mice.

## Materials and Methods

### Animals

All research and animal care procedures were approved by the Garvan Institute / St Vincent’s Hospital Animal Experimentation Ethics Committee and conducted in accordance with the Australian Code of Practice for the Care and Use of Animals for Scientific Purpose. Mice were housed under conditions of controlled temperature (22°C) with a 12h light, 12h dark cycle (lights on at 0700 h). Mice were fed a normal chow diet *ad libitum* (8% calories from fat, 21% calories from protein, 71% calories from carbohydrate, 2.6 kcal/g; Gordon’s Speciality Stock Feeds, Yanderra, New South Wales, Australia). Water was available *ad libitum* for all mice. C57Bl/6JAusb PWCR floxed mice [12] were obtained from Jackson Laboratory and heterozygous mice were bred to homozygosity (PWCR KO) and the additional resulting WT littermates were used as controls. Mice with the selective deletion of PWCR in NPY expressing neurons were generated as described previously [17]. Briefly, floxed PWCR mice (PWCR^lox/lox^) were crossed with a C57Bl/6JAusb transgenic line that drives Cre gene expression under the control of the NPY promoter (NPY^cre/+^) to generate double heterozygous mice (PWCR^lox/+^/ NPY^cre/+^). These mice were then crossed again with PWCR^lox/lox^ mice to generate PWCR^lox/lox^/NPY^cre/+^ mice and controls.

### Bone densitometry and body composition analysis

Bone mineral content (BMC), bone mineral density (BMD) were measured on excised mouse femur bones, using a dedicated mouse dual energy X-ray absorptiometer (DXA) (Lunar Piximus II, GE Medical Systems, Madison WI), as previously described [18].

### Micro Computed Tomography

Skyscan 1174 scanner and analysis software (Skyscan, Aartselaar, Belgium) were used to examine 3-dimensional cortical bone structure and cancellous bone microstructure, as previously described [19]. Following fixation, bone was packed into an enclosed rigid plastic tube filled with 70% ethanol. Distal femora were scanned at 6.2um pixel size. The image projections were reconstructed using NRecon software (Skyscan) and the reconstructed images were aligned using Dataviewer software (Skyscan).

Analysis of the cancellous bone was carried out in 150 slices (0.93mm) selected at 40 slices (0.248mm) from the distal femoral growth plate. Cortical bone analysis was carried out in 150 slices selected at 600 slices (3.72 mm) proximal from the distal femoral growth plate. Whole bone analysis considered all the bone as an ROI. CT-Analyzer software (Skyscan) was used.

For vertebrae, the osteo-cartilage junction at the growth plate was the landmark at each end of the third lumbar vertebral body. Slices between the rostral and caudal landmarks were selected, with 10 slices offset from the rostral and 25 slices offset from the caudal landmark. Vertebral bodies in the selected slices were then separated from the vertebral processes using an elliptical region of interest. This isolated vertebral body was then subdivided into cancellous and cortical parts by manual tracing. Cancellous bone volume was generated for the cancellous region of the third lumbar vertebral body.

### Tissue collection and bone histomorphometry

Upon completion of the study, mice were culled at 16- or 35-weeks of age by cervical dislocation.

For bone analysis both femora were excised and fixed in 4% PBS-buffered paraformaldehyde for 16h at 4°C. The right femora were bisected transversely at the midpoint of the long axis. After dehydration, distal halves were embedded un-decalcified in methyl-methacrylate (Medim-Medizinische Diagnostik, Giessen, Germany).

Sagittal sections of 5 μm thickness were stained and evaluated as previously described [20]. Analysis of cancellous bone volume (BV/TV, %), trabecular thickness (Tb.Th, μm), and number (Tb.N, /mm) was carried out on sections stained with modified von Kossa for mineralized bone. To access bone formation indices, mice were given subcutaneous injections of the fluorescent compound calcein (Sigma Chemical Company, St Louis, USA), 20 mg/kg at 7 and 3 days prior to collection. Mineralizing surface (MS, %) and mineral apposition rate (MAR, μm/d) were measured from unstained sections and bone formation rate (BFR = MS/BS * MAR, μm^3^/μm^2^/d) were calculated. Bone resorption indices—osteoclast surface (Oc.S, %) and number (Oc.N, /mm), were estimated in tartrate-resistant acid phosphatase stained sections [21]. All cancellous measurements were conducted in a sample area bordering the epiphyseal growth plate, beginning 0.25 mm proximal to the mineralization zone to exclude primary spongiosa and extending proximally 4.2 mm, encompassing all the cancellous bone within the cortices, as described previously [22]. Cortical mineral apposition rate was measured at the mid-point of the shaft on the posterior endosteal surface in a region 1000 μm proximal from the mid femora [23].

### Primary osteoblast cultures

Bone marrow mesenchymal stromal cells (BMSCs) were isolated by flushing femur and tibia bones with a 23-gauge needle syringe into complete α-MEM (10% FCS, 100U/ml PenStrep, 1x GlutaMax) and cultured in T-75 flasks. Non-adherent cells were discarded through media changes. BMSCs were seeded at 6 x 10^4^ cells/well in 24-well plates and cultured with 50ug/ml Ascorbic acid and 5mM β-Glycerophosphate for 14 days. Cells were fixed in cold 90% Ethanol for 15 minutes and stained with Alizarin Red for mineralized nodules. Mineralized area was quantified from a color scan of the stained culture plates using Adobe Photoshop CS5.1 (Adobe) for thresholding and ImageJ software (http://rsb.info.nih.gov/ij/) for particle analysis.

### Alkaline phosphatase assay

Alkaline phosphatase activity was determined by using alkaline phosphatase assay kit (Cat# ab83369, ABCAM) following manufacturer’s instructions. In brief, culture medium at day 14 from primary osteoblast cultures of wild-type and PWCR KO was collected. Triplets of 25μl of each sample were added into 96-wells and 50 μl of assay buffer was added, followed by 50 μl of the 5 mM pNPP solution. The reaction was stopped by adding 20 μl of stop-solution for background control. Standard curves were generated as manufacturer’s guide. The alkaline phosphatase enzyme activity of each sample was calculated based on the comparison between standard curve and sample curve, and was shown as Unit/ml (U/ml).

### Analysis of gene expression

RNA was isolated from cleaned tibiae using TRIzol reagent as per the manufacturer’s instructions. Real-time polymerase chain reaction was used to determine gene expression of osterix, osteocalcin, Runx-2, alkaline phosphatase, receptor activator of nuclear factor kappa-B ligand (Rankl), osteoprotegerin (Opg), and the housekeeping genes β-actin, *Gapdh* (Taq-Man Gene Expression assays for Runx-2, alkaline phosphatase, Rankl, Opg from Applied Biosystems, Foster City, CA). Primer sequences for osterix, osteocalcin, Gapdh are as follows: osteocalcin (sense: 5′-TTCTGCTCACTCTGCTGACCCT-3′, antisense:5′-CCTGCTTGGACATGAAGGCTT-3′), osterix (sense: 5′-AGAGGTTCACTCGCTCTGACGA -3′, antisense: 5′-TTGCTCAAGTGGTCGCTTCTG-3′), Gapdh (sense: 5′-GCATCTCCCTCACAATTTCCA-3′, antisense:5′-GTGCAGCGAACTTTATTGAT GG-3′). cDNA synthesis was performed with 0.5–1 μg of total RNA with oligo(dT)20 by using the SuperScript III First-Strand Synthesis System for reverse transcription-PCR (Invitrogen). Real-time polymerase chain reaction reactions were performed using the TaqMan Universal PCR master mix and Power SYBR Green PCR Master Mix (Applied Biosystems) in an ABI Prism 7900 HT sequence detector (Applied Biosystems).

### Primary osteoclast cultures

Mouse bone marrow cells were used for the osteoclast formation assay. Briefly, 10-week-old female wild-type and PWCR KO mice were sacrificed by cervical dislocation, and marrow was flushed from tibias supplemented with 10% FBS, 150 μg/ml penicillin, 125 μg/ml streptomycin, 1.25 μg/ml Fungizone. Cleaned tibias were used for RNA isolation as described below.

The bone marrow cell suspension was aspirated through a 21-gauge needle and filtered with a 100-μm-pore-size cell strainer filter (Falcon/Becton Dickinson, Franklin Lakes, NJ). Cells were then washed twice in culture medium and centrifuged for 10 min at 200×*g*, and 1 × 10^5^cells/cm^2^ were plated in 96-well flat-bottom tissue culture-treated plates (Costar, Cambridge, MA, USA) in 150 μl of culture medium containing 30 ng/ml recombinant murine M-CSF (R&D Systems, Minneapolis, MN, USA) and 20 ng/ml recombinant murine RANKL (RANKL-TEC, R&D Systems, Minneapolis, MN, USA). Culture medium was refreshed after 3 days. After 7 days of co-culture, cells were fixed in 4% formaldehyde in PBS for 10 min. Fixed cells were washed with PBS and stained for tartrate-resistant acid phosphatase (TRACP) according to the manufacturer’s instructions (Sigma). The number of TRACP-positive multinucleated (three or more nuclei per cell) and mononuclear cells were counted with a Leica DM IL microscope (Leica, Wetzlar, Germany) equipped with a ×20 objective.

### Statistical analysis

Statistical analyses were performed using GraphPad Prism 6.00, statistically significant values were those with p values less than 0.05. All data are expressed as a mean ± standard deviation (SD). Within gender, across genotype comparisons were carried using student’s T test.

## Results

### Lack of PWCR in mice reduces bone mass specifically affecting cortical bone

**Fig 1:**
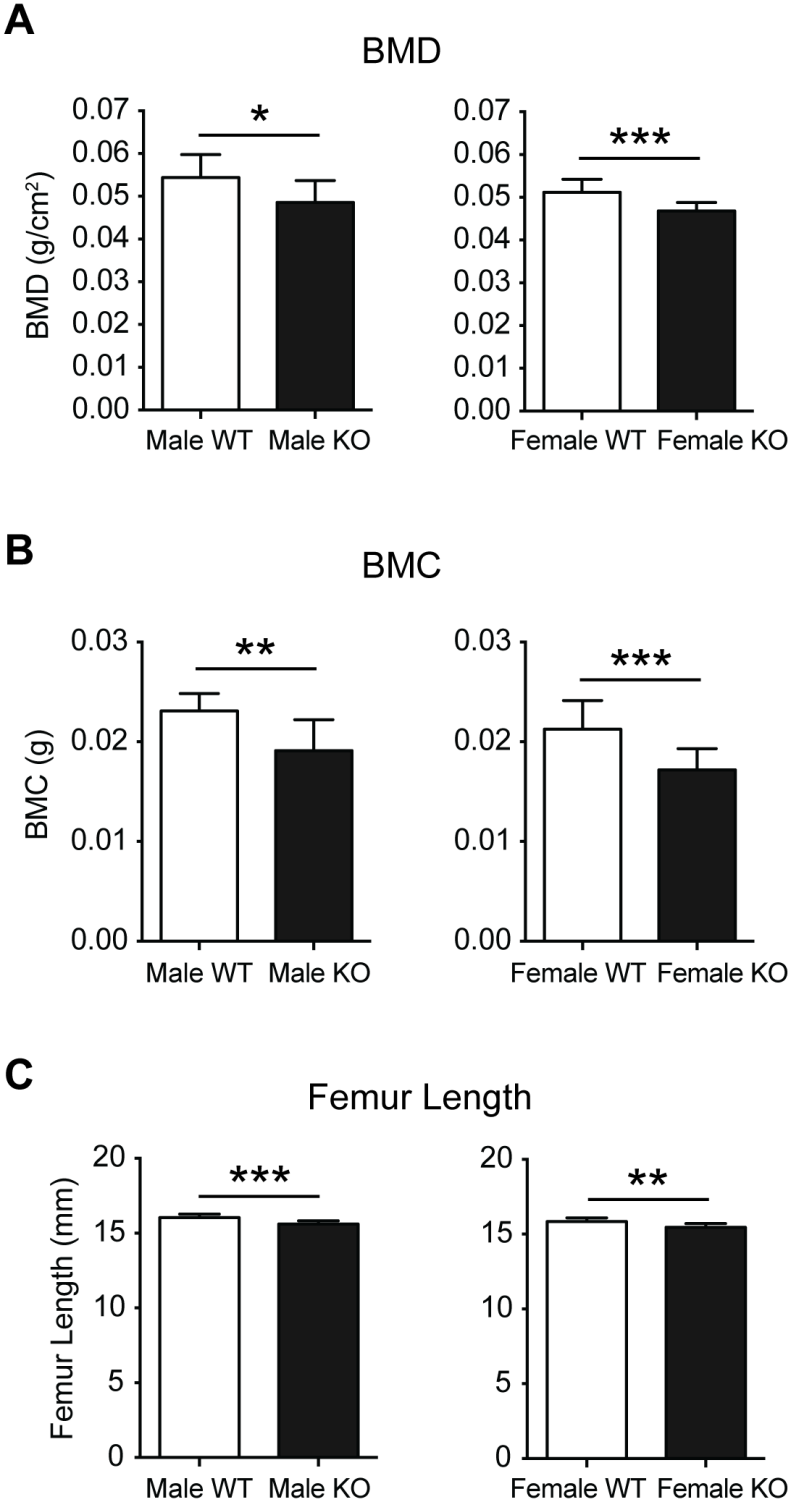
Whole bone analysis of PWCR KO mice. Femurs from 16 week old male and female PWCR KO and WT mice were dissected and analysed by DXA and measured for length. A.) BMD; B.) BMC and C.) femur length. n = 7–10 represented as mean ± SD. * p-value<0.05, ** p-value<0.01, *** p-value<0.001.

DXA analysis of excised femurs from 16 week old male and female PWCR KO mice revealed a marked bone phenotype in both genders, with femoral BMD and BMC significantly reduced compared to age- and sex-matched wild type mice (WT) (Fig 1A and 1B). These changes were consistent with a reduction in femur length (Fig 1C) in both genders of PWCR KO mice.

**Fig 2:**
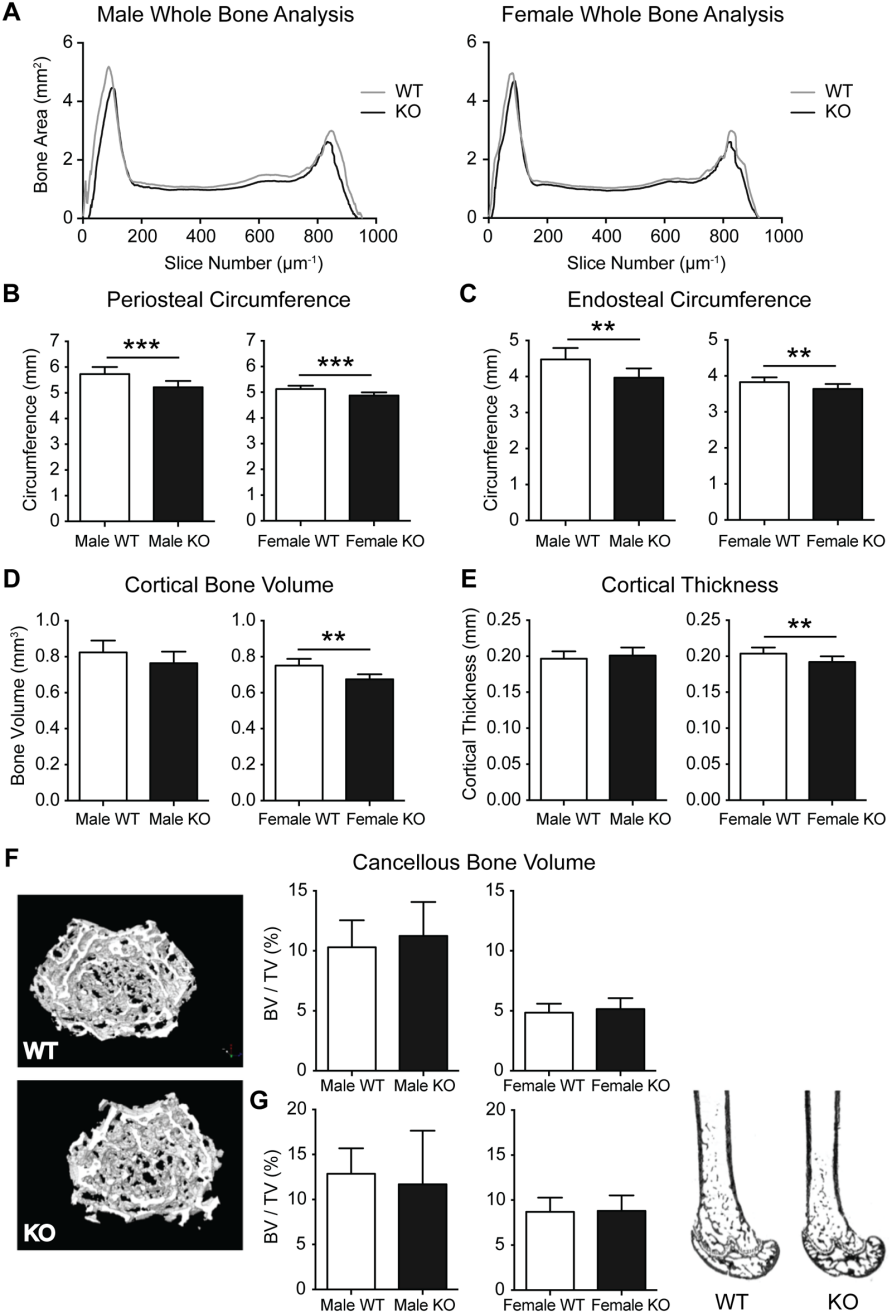
Cortical bone analysis of PWCR KO mice. A.) Whole bone analysis of male and female femurs of PWCR KO and WT mice using micro CT. Micro CT analysis of cortical bone on a standardised region of the femur diaphysis :- B.) periosteal circumference; C.) endosteal circumference; D.) cortical bone volume; and E.) cortical thickness. F.) Micro CT analysis of cancellous bone of the distal femur. G.) Representative images and histomorphometric analysis of sagittal femoral cancellous bone volume. n = 7–10 represented as mean ± SD. * p-value<0.05, ** p-value<0.01, *** p-value<0.001.

In order to investigate whether the bone phenotype extended beyond a reduction in bone length; the entire femur was scanned by μCT, illustrating a generalized reduction in cross-sectional bone area in the diaphysis of KO femurs (Fig 2A). Detailed μCT analysis demonstrated reductions in periosteal and endosteal perimeter at the diaphysis in both sexes (Fig 2B and 2C). The reduced cortical dimensions were accompanied by reduced cortical bone volume and cortical thickness in PWCR KO femurs (Fig 2B and 2C). In contrast, analysis of cancellous bone by μCT in intact femurs (Fig 2F), and analysis of von kossa stained sagittal femoral sections by histomorphometry (Fig 2G), revealed no difference in the micro-architecture of cancellous bone between WT and PWCR KO.

**Fig 3:**
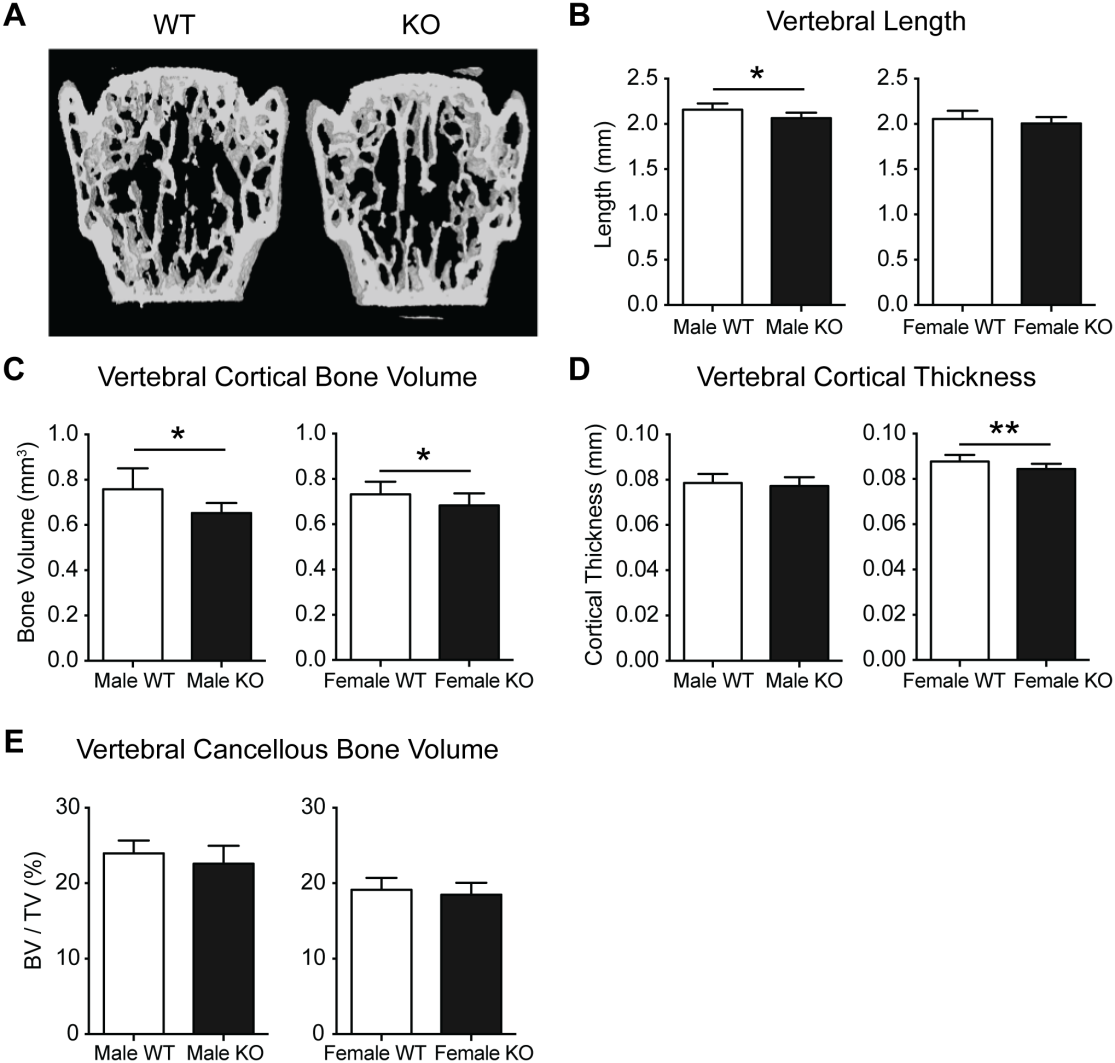
Lumbar vertebral bone analysis of PWCR KO mice. A.) Representative images and micro CT analysis of L3 vertebral bone :- B.) Vertebral length; C.) cortical bone volume; D.) vertebral cortical thickness; E.) cancellous bone volume. n = 7–10 represented as mean ± SD. * p-value<0.05, ** p-value<0.01.

To determine whether the diminished cortical production in PWCR KO mice was a generalised effect, we examined the axial skeleton. Vertebral length (L3 vertebrae) was reduced in male PWCR KO mice, with a similar but non-significant reduction in females (Fig 3A and 3B). Recapitulating the femoral changes, cortical bone volume was reduced in PWCR KO relative to WT mice, with cortical thickness significantly reduced in female KO mice only (Fig 3C and 3D). Vertebral cancellous bone volume was unchanged between WT and KO mice (Fig 3E).

### Lack of PWCR reduces bone formation and osteoblast differentiation, without alterations in osteoclastogenesis

**Fig 4:**
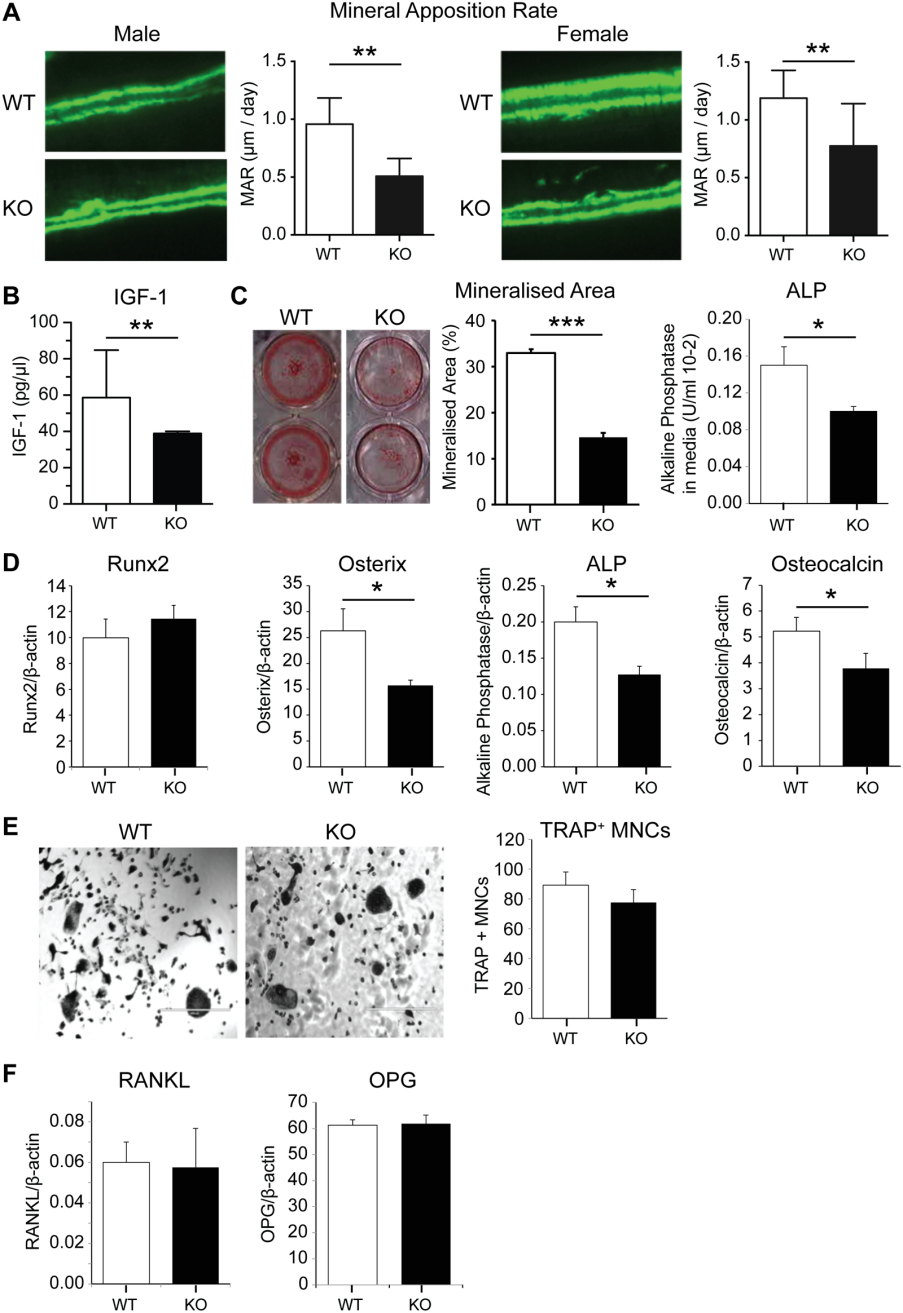
Bone cell activity and expression analysis of PWCR KO mice. A.) Bone histomorphometric analysis of double fluorochrome labelled femur sections showing Mineral Apposition Rate (MAR). n = 6–10 represented as mean ± SD. B.) Serum IGF-1. C.) Alizarin red staining in bone marrow stromal cells (BMSCs) cultured from WT and PWCR KO femur and tibia bones, after 14 days in osteogenic medium; Mineralized area; Alkaline phosphatase activity in culture media. D.) Expression of osteoblast markers in bone isolates from biological replicates of 16 week old mice:- Runx2; Osterix; Alkaline phosphatase; Osteocalcin. E.) Osteoclast cultures from marrow of WT and PWCR KO mice:- representative images; TRAP+ multinuclear cell number. F.) RANKL and OPG expression in bone isolates from biological replicates of WT and PWCR KO mice. n = 4 represented as mean ± SEM. * p-value<0.05, ** p-value<0.01, *** p-value<0.001.

The reductions in bone length and size indicate the existence of a developmental effect occurring in PWCR KO mice, consistent with the reduced stature evident in PWS. However, given the ongoing skeletal issues in PWS, we sought to determine whether the skeletal effects of PWCR extended beyond growth. In order to compare the bone cell activity in adult mice with the architectural changes evident in PWCR KO mice, we examined bone formation indices in a region corresponding to previous μCT analysis in the femora. This region constitutes part of the modelling drift responsible for dimensional changes in mature lone bones [24], and as such in sagittal sections this surface is consistently covered by bone formation. Importantly, analysis of double fluorochrome labelling in 16 week old mice, showed a marked decrease in periosteal MAR in both genders of PWCR KO mice (Fig 4A). This result indicated that loss of the PWCR was capable of altering bone formation in mature mice, and illustrates an effect beyond regulation of developmental processes. Consistent with the observed reductions in growth and bone size, ongoing anti-anabolic actions, and previous publications [12], serum IGF-1 levels were markedly reduced in PWCR KO compared to wild type (Fig 4B). To investigate whether the deficiencies in bone mass were due to an intrinsic defect in osteoblast activity, PWCR KO bone marrow stromal cells (BMSC) were differentiated to osteoblasts *ex vivo* and their properties investigated. Alizarin red staining, as a marker of mineralisation was reduced at day 21 of culture in PWCR KO osteoblast cultures compared to WT, alkaline phosphatase activity in the culture medium was reduced at day 14 (Fig 4C). Gene expression for markers of mature osteoblasts were reduced in cortical bone from PWCR KO mice, with Osterix, alkaline phosphatase and osteocalcin reduced, albeit with unchanged Runx2 (Fig 4D). The osteoblast-specific nature of the PWCR KO phenotype was determined by examining osteoclastic development *in vitro*. Osteoclast cultures from PWCR KO bone marrow monocytes (BMMs) showed equivalent production of TRAP+ multinucleated cells after RANKL treatment compared to WT (Fig 4E), indicating no effect upon osteoclastic potential by Snord116. The osteoblastic stimulation of osteoclastogenesis was also unaltered in PWCR KO mice, with RANKL and OPG production by osteoblasts not different between PWCR KO and WT mice (Fig 4F).

### PWCR knockout bone phenotype persists with age

**Fig 5:**
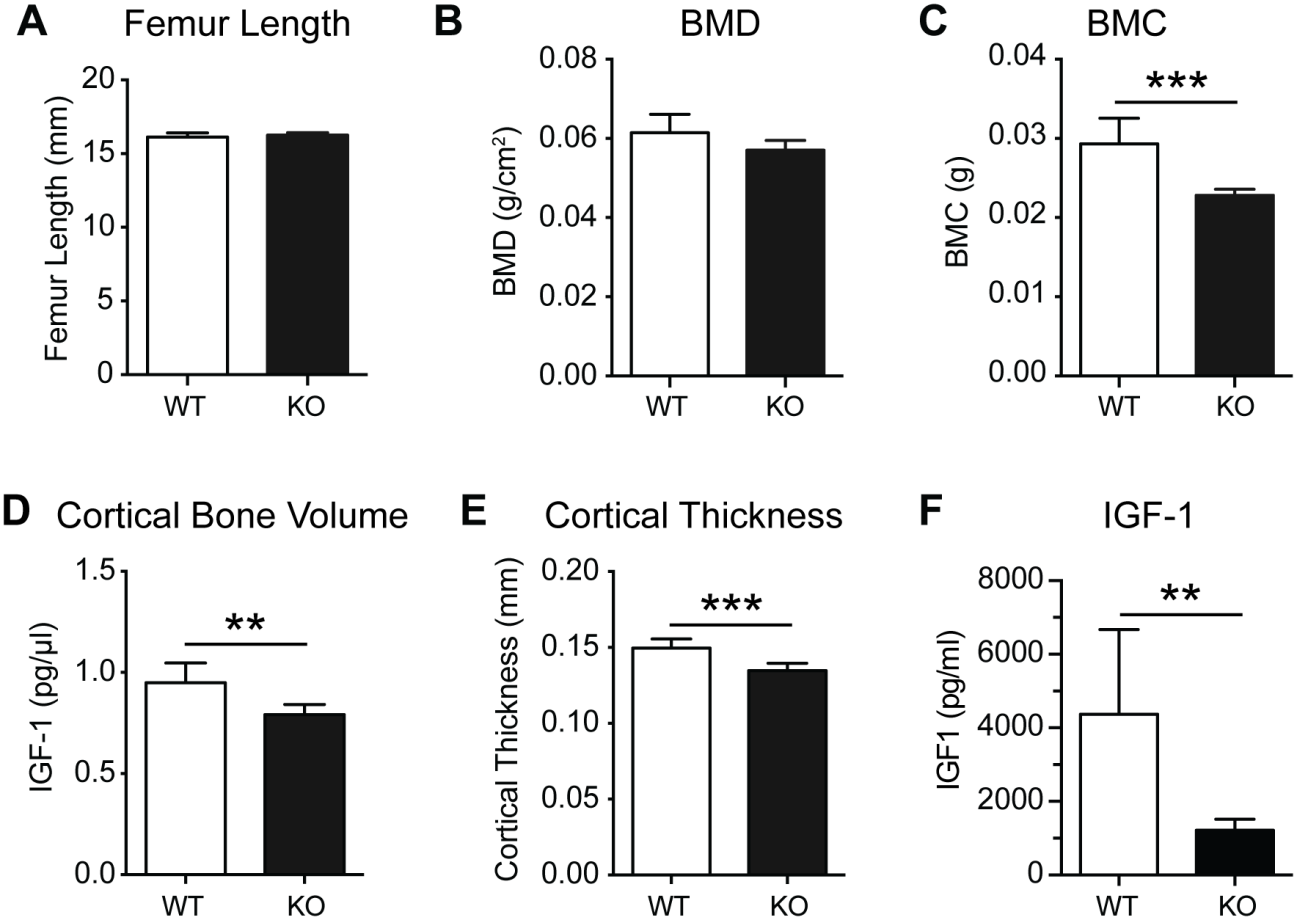
Bone characterisation of aged PWCR KO mice. A.) Femur length of 35 week old male WT and PWCR KO mice; B.) BMD and C.) BMC measured by DXA of isolated femurs; Three-dimensional μCT analysis of cortical and cancellous bone D.) cortical bone volume; E.) cortical thickness; F.) IGF-1 n = 6 represented as mean ± SD. ** p-value<0.01, *** p-value<0.001.

Since PWS is characterised by impaired early development, and having demonstrated impaired bone formation after skeletal maturity, we examined whether this would be corrected in later life. Interestingly, in contrast to 16-week-old mice, femur length was not different between male WT and KO mice at 35 weeks of age (Fig 5A), consistent with the lack of growth plate closure in mice. Importantly, despite the correction of femur length, 35-week-old KO mice retained a significant reduction in femoral BMC (Fig 5C), although DXA analysis revealed similar BMD as that in WT mice (Fig 5B), suggesting reduced bone size in KO mice. Indeed, similar to the 16-week-old bone phenotype, cortical bone volume and cortical thickness were significantly reduced in the femurs of 35-week-old PWCR KO mice (Fig 5C). Importantly, IGF-1 was still markedly reduced in PWCR KO mice at this age (Fig 5E). Similarly to younger mice, cancellous bone volume was not different between 35 week old PWCR KO and WT mice (data not shown). Thus loss of PWCR expression results in long-term deficits in cortical bone accrual.

### Selective deletion of the PWCR only in NPY neurons replicates the PWCR KO bone phenotype

**Fig 6:**
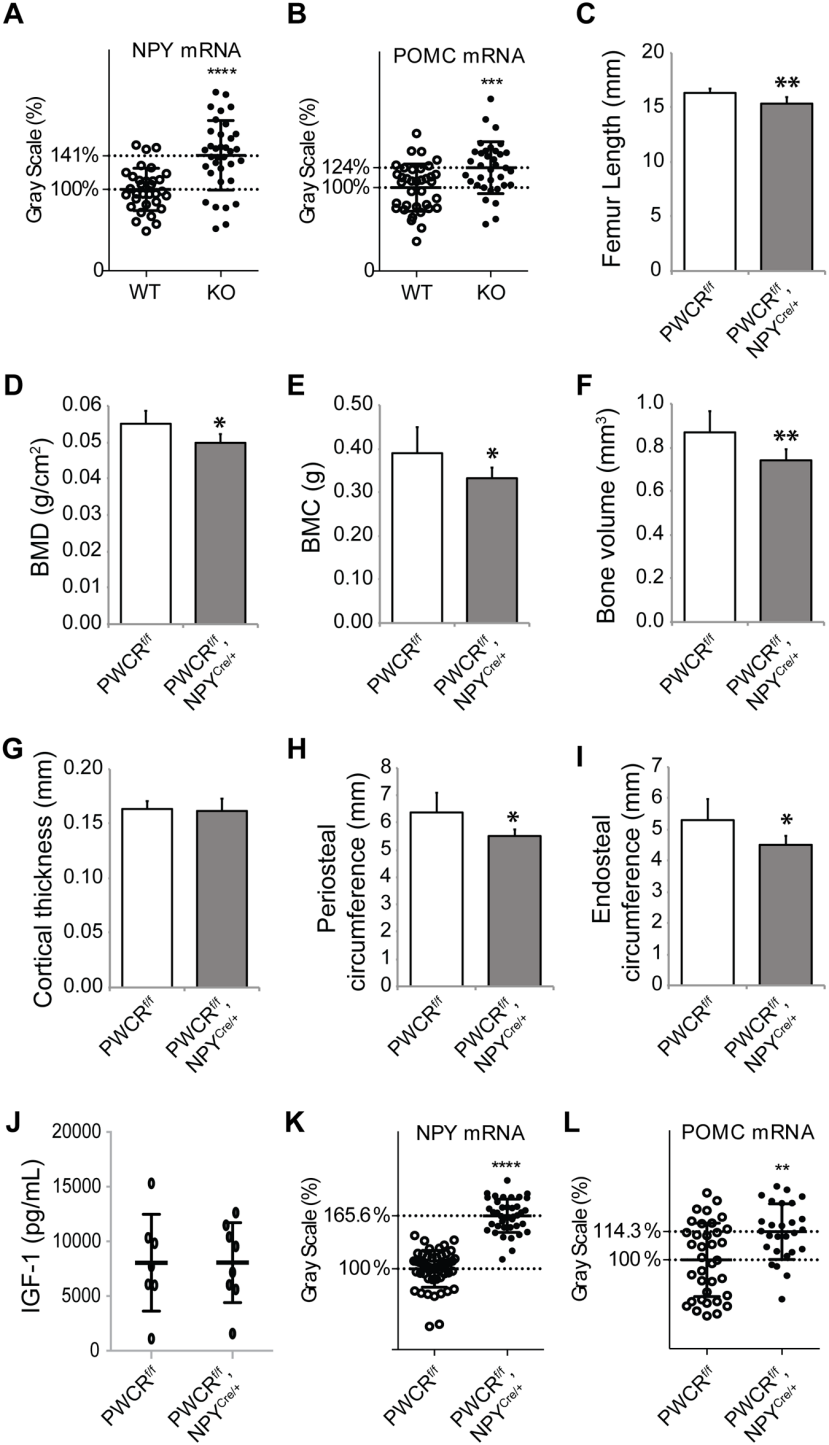
Selective deletion of PWCR only in NPY neurons. In situ hybridisation for A.) NPY and B.) POMC expressions in arcuate nucleus of WT and PWCR KO mice. Bone phenotype analysis of PWCR^f/f^, NPY^Cre/+^ and PWCR^f/f^ control mice: C.) femur length; D.) whole body BMD; E.) whole body BMC measured by DXA; F.) cortical bone volume; G.) cortical thickness; H.) periosteal circumference and I.) endosteal circumference measured by μCT. n = 6–8 represented as mean ± SD using 16 week old male mice. J.) Serum IGF-1 levels and *in situ* hybridisation for K.) NPY and L.) POMC in the arcuate nucleus of PWCR^f/f^, NPY^Cre/+^ and PWCR^f/f^ control mice. n = 4–6 represented as mean ± SD using 16 week old male mice. * p-value<0.05, ** p-value<0.01, *** p-value<0.001.

As Snord116 expression is confined to the brain [25, 26], the reduction in bone formation most likely reflects alterations in signalling from the central nervous system. Neuropeptide Y (NPY) is a classic neuronal regulator of energy homeostasis and has also been shown to play an important role in the regulation of bone formation [18]. Therefore, we investigated the mRNA expression levels of NPY and the counter-regulatory proopiomelanocortin (POMC) in the arcuate nucleus of PWCR KO and WT mice by *in situ* hybridisation. Interestingly, the expression levels of both NPY and POMC were significantly increased in the PWCR KO mice (Fig 6A and 6B). This disruption to the normally observed counter-regulatory pattern of NPY and POMC mRNA expression suggests that the inhibiting projections from NPY neurons to POMC neurons do not function in the normal way in the absence of the PWCR. Previous studies elevating NPY expression solely in the arcuate nucleus have demonstrated a marked suppression of osteoblast activity on cortical bone surfaces [18].

In order to determine whether these neuronal alterations are driving the bone phenotype of PWCR KO mice, we investigated the bone phenotype of male mice with a specific deletion of the PWCR only in NPY neurons (PWCR^f/f^, NPY^Cre/+^) [17]. As shown in Fig 6C–6E, similar to PWCR KO mice, PWCR^f/f^, NPY^Cre/+^ mice display significantly reduced whole body BMD, BMC and femoral length compared to controls. Furthermore, selective deletion of the PWCR from NPY neurons resulted in a cortical bone phenotype similar to that of germline PWCR KO mice with a significant reduction in cortical bone volume as well as periosteal and endosteal perimeter with no change in cortical thickness (Fig 6F–6I).

In order to confirm and further investigate the direct influence of the PWCR on NPY neurons we measured serum IGF-1 levels and performed *in situ* hybridisation. Although serum IGF-1 levels were unchanged (Fig 6J), both arcuate nuclei NPY and POMC mRNA expression were significantly upregulated (Fig 6K and 6L) identical to the global deletion model. Taken together, these data indicate a critical role of the PWCR in NPY neuronal function and the regulation of bone mass.

## Discussion

In this study we demonstrate the critical role of the PWCR which contains the Snord116 cluster in the regulation and maintenance of bone mass, and thereby identify the first pathway from the brain to bone mediated by a region encoding non-translated RNAs, and suggest a putative role for this in human disease. The analysis of mice deficient in the PWCR revealed a skeletal phenotype that shows strong parallels with that found in humans with PWS. In particular, lack of the PWCR led to a reduction in the length of long bones (femur) and vertebral bodies in skeletally mature animals, however, this was corrected when mice reached an older age, consistent with non-closure of growth-plates in rodents. Importantly, both mature and aged mice displayed a significant reduction in cortical bone formation and cortical bone size, indicating a prolonged action of the PWCR in skeletal homeostasis. These changes were coincident with reduced serum IGF-1 levels, indicating a long-term disruption of the somatotropic axis. Consistent with the restriction of Snord116 expression to the brain; we identify the PWCR in NPY neurons as critical to the pathway to bone, highlighting the central nature of the PWCR signalling axis. PWCR deletion led to an upregulation in both NPY and POMC expression levels in the arcuate nucleus, a surprising finding given that NPY and POMC normally show opposing expression levels in response to situations of positive or negative energy balance. Selective deletion of the PWCR only in NPY neurons was sufficient to replicate the bone phenotype of germline PWCR KO mice, without any alteration in circulating IGF-1 levels, as well as the deregulated simultaneous upregulation of both NPY and POMC levels. This model also has similarities to the skeletal phenotype of mice with specific overexpression of NPY in the arcuate [27]. In this manner, the PWCR KO mice provide a novel model to study the skeletal defects in PWS through both endocrine and neural pathways. Moreover, the identification of a region encoding non-translated genes regulating bone mass reveals a new level of complexity involved in the central regulation of bone mass.

In patients with PWS, short stature and reduced BMD are commonly observed, along with growth hormone deficiency, [3, 14], however, the precise mechanism involved in these changes has yet to be defined. The short stature and low BMD have been linked to a lack of sufficient GH production [13]. GH supplementation remains one of the front line therapies for both body composition and skeletal abnormalities in PWS, most commonly in infants/children but also in adults [28]. However, GH treatment on PWS children had no long-term effects on BMD, indicating that other factors are also involved [13]. The reduction in the downstream effector of GH, IGF-1, observed in PWCR KO mice in the current, and previous studies [12] is consistent with the GH deficiency observed in PWS and highlights the potential to better define the disease process in PWS. Mouse genetic models have produced a detailed understanding of the effects of IGF-1 and GH deficiency on the skeleton. GHRH receptor, GH receptor and liver specific IGF-1 (LID) knockout mouse models reduce the circulating pool of IGF-1 but not skeletally produced IGF-1, leading to a reduction of cortical parameters but leaving cancellous bone unchanged [29]. This scenario is parallel to what appears to be evident in the PWCR KO mice, with alterations of central PWCR expression in the hypothalamus disrupting the central somatotropic axis, reducing circulating IGF-1 levels. In addition, while not as similar as the present model, GH receptor KO and IGF-1 KO mice also show significantly reduced periosteal mineral apposition rates [30, 31]. It is important to note that PWCR KO mice are not complete photocopies of PWS, particularly in body composition. Despite hyperphagia, KO mice are leaner and lighter at younger ages, although this is corrected with age [12]. One other hormone that has been implicated in the phenotype of PWS is ghrelin. However, no obvious changes in ghrelin serum levels have been observed in our model [17], suggesting this hormone seems less likely to play a role in the observed bone phenotype of PWCR deficient mice. However, the consistency of the skeletal changes in the PWCR KO mouse represent an important opportunity to increase our understanding of PWS, and also highlights the potential complexity of the regulatory processes involved in the disease aetiology.

The reduction in BMD in PWCR KO mice was attributable to a cortical-specific phenotype evident in both axial and appendicular bone. In the femur, PWCR deficiency reduced endosteal and periosteal circumferences, with reduced cortical bone volume also evident in the vertebrae. These reductions in dimension were retained even when linear differences were absent in aged mice. Interestingly, cancellous bone mass and architecture were normal in KO mice. The growth retardation indicated by reduced femoral and vertebral length at 16 weeks contributed to the observed deficits in cortical bone mass during early development of PWCR KO mice. However, the reduction in mineral apposition in aged mice (35 weeks), when length differences were absent, implies that the PWCR continues to exert effects on bone homeostasis beyond development. Femur length was corrected with age, which is likely associated with the lack of closure of murine compared to human growth plates. However, the low bone mass phenotype was maintained, and demonstrated signs of exacerbation, with cortical area and thickness significantly reduced in male PWCR KO mice at 35 weeks, but not at 16 weeks. Thus PWCR action is required for both development and maintenance of bone mass throughout life. That such a region containing non-translated RNAs, expressed solely within the brain and from an imprinted region such as the PWCR, can exert substantial and life-long effects upon bone homoeostasis illustrates a new level of complexity of skeletal regulation.

The hypothalamus is known to play a major role in the development of pathologies in PWS. Snord116 is exclusively expressed in the brain of mice, with high expression in the hypothalamus [25, 26]. Given the known role of central NPY in the regulation of bone mass and the observed upregulation of NPY and POMC expression in the arcuate nucleus of PWCR KO mice, mice with selective deletion of the PWCR only in NPY neurons were investigated [17]. These mice almost exactly replicated the alterations in bone mass, femoral length, and NPY/POMC expression levels seen in the germline PWCR KO mice, without alterations in IGF-1 levels. This highlights the importance of this population of neurons and their critical role in the bone phenotype of PWS. The changes seen in NPY and POMC mRNA, which are both upregulated in these mice, are surprising since they normally show opposing expression levels in response to situations of positive or negative energy balance. It has been clearly established that NPY has the dominant role in this counter regulatory process and high NPY levels would normally cause the down-regulation of POMC mRNA. The fact that this is not the case in this model suggests a vital connection or functional response is missing when there is a lack of the PWCR. Mice lacking NPY have an anabolic bone phenotype in both cortical and cancellous compartments characterised by elevated bone formation, whilst in contrast, viral-mediated elevation of hypothalamic NPY levels in wildtype mice results in a reduction in bone formation despite increased body weight [18]. Mechanistically, NPY has been shown to exert these effects through regulating the differentiation of osteoblast precursors as well as the action of mature osteoblasts [21, 32]. The fact that the PWCR deletion led to upregulated NPY levels and changes in cortical bone mass and bone formation without changes in cancellous bone mass, may be due to the deregulated counter-regulatory response between NPY and POMC expression levels, but also highlight that direct neuronal actions may contribute to the observed bone phenotype.

Little is known about Snord116 other then that it is a C/D box snoRNA exclusively expressed in mouse brains [25]. These C/D box snoRNAs are involved in methylation by interacting, through base pairing, with ribosomal RNA (rRNA) or small nuclear RNA (snRNA) [33]. Our appreciation of the important role non-translated species play in physiological processes is in its infancy, particularly with regard to functional studies. Moreover, our understanding of RNA control of bone is confined to microRNA species within the bone micro-environment [34]. Thus, the PWCR model may represent the first non-local, non-translated regulator of bone to be identified. The growing premise that Snord116 regulates the somatotropic axis as well as neuronal output to peripheral tissues may have important implications for PWS. The pituitary and hypothalamus are the centres of the growth hormone axis and are thought to be affected in PWS, and GH supplementation is a front line therapy [35, 36]. Indeed, Snord116 is highly expressed in these regions and knockout mice display other characteristics of pituitary-hypothalamus dysfunction such as delayed sexual maturation in female mice and hyperphagia [12]. Interestingly, it was reported that PWCR KO mice have normal pituitary and normal numbers of GH expressing neurons, indicating a defect in the hypothalamic control of GH release [12]. This is reinforced by the reduced IGF-1 levels in this study.

However is also important to keep in mind that Snord116 is being produced as a cleavage product out of intronic sequences of the large SNURF-SNRPN transcripts and the deletion of the PWCR removes the Snord116 cluster as well as approximately 150kb of genomic sequence, including some of the exons of the SNURF-SNRPN transcripts. It is therefore possible that alterations to the SNURF-SNRPN or long U-Ube3A antisense transcripts contained within this region could have contributed to an altered function of NPY neurons.

In conclusion, this study provides evidence that regulation of the PWCR also containing the non-translated snoRNA, Snord116 in the hypothalamus plays a marked role in development and maintenance of cortical bone mass and structure, through regulation of longitudinal growth and periosteal and endosteal bone formation. Bone homeostasis is disrupted by deletion of the PWCR, and this can be traced to altered actions in NPY neurons, supporting brain-bone regulation by non-coding RNAs and clarifying the complex phenotype of PWS. As such, this represents the first report of a central, RNA-mediated pathway to bone. Moreover, Snord116 expression plays a causal role in the bone phenotype of PWS individuals. While the germline knockout model does not completely reproduce the human PWS, it does highlight the significant contribution of the PWCR to the observed human phenotype as also seen in some behavioural aspects [37]. The PWCR KO mouse represents an important tool for study of the complex aetiology of PWS.
